# Defective chromatin recruitment and retention of NHEJ core components in human tumor cells expressing a Cyclin E fragment

**DOI:** 10.1093/nar/gkt812

**Published:** 2013-09-09

**Authors:** Payel Chatterjee, Dragos Plesca, Suparna Mazumder, Jean Boutros, Steven M. Yannone, Alexandru Almasan

**Affiliations:** ^1^Department of Cancer Biology, Lerner Research Institute, Cleveland Clinic, Cleveland, OH 44195, USA, ^2^School of Biomedical Sciences, Kent State University, Kent, OH 44234, USA, ^3^Department of Chemistry, Cleveland State University, Cleveland, OH 44115, USA, ^4^Life Sciences Division, Lawrence Berkeley National Laboratory, Berkeley, CA 94720, USA and ^5^Department of Radiation Oncology, Taussig Cancer Institute, Cleveland Clinic, Cleveland, OH 44195, USA

## Abstract

Exposure to genotoxic agents, such as ionizing radiation (IR), produces double-strand breaks, repaired predominantly in mammalian cells by non-homologous end-joining (NHEJ). Ku70 was identified as an interacting partner of a proteolytic Cyclin E (CycE) fragment, p18CycE. p18CycE endogenous generation during IR-induced apoptosis in leukemic cells and its stable expression in epithelial tumor cells sensitized to IR. γH2AX IR-induced foci (IRIFs) and comet assays indicated ineffective NHEJ DNA repair in p18CycE-expressing cells. DNA pull-down and chromatin recruitment assays revealed that retention of NHEJ factors to double-strand breaks, but not recruitment, was diminished. Similarly, IRIFs of phosphorylated T2609 and S2056-DNA-PKcs and its target S1778-53BP1 were greatly decreased in p18CycE-expressing cells. As a result, DNA-PKcs chromatin association was also increased. 53BP1 IRIFs were suppressed when p18CycE was generated in leukemic cells and in epithelial cells stably expressing p18CycE. Ataxia telangiectasia mutated was activated but not its 53BP1 and MDC1 targets. These data indicate a profound influence of p18CycE on NHEJ through its interference with DNA-PKcs conformation and/or dimerization, which is required for effective DNA repair, making the p18CycE-expressing cells more IR sensitive. These studies provide unique mechanistic insights into NHEJ misregulation in human tumor cells, in which defects in NHEJ core components are rare.

## INTRODUCTION

Exposure to genotoxic agents, such as ionizing radiation (IR), can induce various forms of DNA damage, amongst which the most lethal ones are the DNA double-strand breaks (DSBs); if left unrepaired, they may lead to cell death (1). IR-triggered DSBs are prevalently repaired by non-homologous end-joining (NHEJ) in mammalian cells (2,3). The core components of NHEJ are the Ku70-Ku80 heterodimer, DNA-PK catalytic subunit (DNA-PKcs), XRCC4, Ligase IV and accessory factors, such as XLF/Cernunnos (3–5) and 53BP1 (6). The Ku70/80 complex detects DSBs and recruits DNA-PKcs, which gets activated by dimerization and creates the stage for assembly of other NHEJ factors (7,8). The role of DNA-PKcs has been implied by phosphorylation of a number of pivotal NHEJ repair proteins, such as Artemis and XRCC4 (9–11). Thus, DNA ends are processed by Artemis and several DNA polymerases, with DNA-PKcs autophosphosphorylation leading to its dissociation from DNA. As the final step, DNA ligation is completed by XRCC4 and DNA ligase IV (7). 53BP1 is a critical transducer of the IR-induced DNA damage signal (6,12), which promotes NHEJ by repressing homologous recombination (HR) (13). 53BP1 is phosphorylated on several residues in response to radiation, causing cells to develop distinct IR-induced foci (IRIFs) of total and phosphorylated forms of 53BP1.

The principal nuclear serine/threonine kinase involved in NHEJ is DNA-PK, which contains a catalytic subunit and a regulatory subunit, Ku70-80 (8). Once recruited to DNA ends, activated DNA-PKcs autophosphorylates at numerous residues, the best studied being those in the ABCDE and PQR clusters (14). Several reports suggest that phosphorylation of T2609 present in the ABCDE cluster (15,16), either by *trans*-autophosphorylation (14) or by Ataxia telangiectasia mutated (ATM) (17), is critical for NHEJ, as it regulates the dissociation of DNA-PKcs from DNA ends (7). In response to IR, DNA-PKcs is autophosphorylated at the S2056 site in the PQR cluster (18,19). Inhibition of phosphorylation at S2056 in the PQR cluster in ABCDE phospho-mutant cells enhances HR, indicating the critical role of the S2056 phophorylation site in suppressing HR and supporting NHEJ (19). Cells containing DNA-PKcs with site-directed mutations in the S2056 and T2609 sites by amino acid replacement exhibit hyper-radiosensitivity (20) comparable with DNA-PKcs-deficient cells, thus reinforcing the importance of functional DNA-PKcs in the radiation response (1,3).

An 18-kDa fragment of Cyclin E (p18CycE), generated by caspase-dependent proteolytic cleavage, was first discovered in leukemic cells undergoing apoptosis (21). In the cytosol, p18CycE binds to Ku70 and disrupts the Bax-Ku70 association, leading to Bax activation and apoptosis (22). The consequences of p18CycE binding to Ku70 in the nucleus have not been examined. In contrast to leukemic cells, solid tumors have not been found to produce p18CycE on irradiation or treatment with other apoptosis-inducing chemotherapeutics. Ectopic expression of p18CycE in these non-leukemic cell lines confers a radiosensitive phenotype (22), suggesting that p18CycE may affect DNA damage and its repair.

On investigation of the role of p18CycE in DNA repair, we found that its expression, whether achieved endogenously (as in leukemic cells) or ectopically (as in epithelial tumor cells), leads to persistent DNA damage following irradiation and reduced cell survival. In p18CycE-expressing cells, the NHEJ core factors are recruited to DSBs after DNA damage but are unable to be retained there long enough for successful DNA repair. Ineffective autophosphorylation of DNA-PKcs leads to its persistence on chromatin in p18CycE-expressing cells, resulting in diminished recruitment of other DNA repair proteins, such as 53BP1. These findings uncover a novel mechanism for inactivation of NHEJ that can impact the survival of human tumor cells.

## MATERIALS AND METHODS

### Cell culture and treatment

Human epithelial tumor (kidney HEK 293T and prostate cancer C4-2) and leukemic (MOLT4 and Jurkat) cells were cultured as described previously (22,23) in Dulbecco’s modified Eagle’s medium and RPMI-1640 medium, respectively, with 10% fetal bovine serum (Atlanta Biologicals, Norcross, GA, USA), l-glutamine and antibiotic-antimycotic (Invitrogen, Carlsbad, CA, USA) in a humidified incubator at 37°C and 5% CO_2_. Cells stably expressing p18CycE were generated for HEK 293T cells as described (24) and for C4-2 prostate cancer cells after their transfection with hemagglutinin (HA) p18CycE using the FuGENE HD transfection reagent (Promega, Madison, WI, USA). Cells were seeded the day before transfection in antibiotic-free media to achieve ∼80% confluency by next day. On the day of transfection, in one tube, 1.5 µg of DNA (six-well plate or 35-mm dish) was mixed in Optimem (Invitrogen); in another tube, FuGENE was mixed with Optimem in a 1:5 ratio, with both being incubated for 5 min. Then both DNA and FuGENE HD were mixed together and incubated for another 15 min. Finally, the mixture was added to the cells and incubated for 30 h after which fresh media was added to the cells. Cells were then selected with G418 (1 mM; Invitrogen) for stable expression of p18CycE. For transient transfection, a similar protocol was used, except Lipofectamine (Invitrogen) was substituted for FuGENE HD in a 2:5 ratio. For EGFP transfection, the vector backbone was pEGFP-C1 (Clontech, Mountain View, CA, USA). IR was administered using a conventional cesium-137 -irradiator (JL Shepherd Associates, San Fernando, CA, USA), at a dose rate of 146 cGy/min, as described.

### Colony formation assay

For clonogenic assay, 500 cells/60 mm dish were seeded 1 day before the treatment. The next day, different doses of radiation were delivered. After 14 days, cells were stained with 0.1% crystal violet and colonies, with >50 cells, and were scored by an alpha image analyzer (Alpha Innotech Corp.). For clonogenic assay of MOLT4 and Jurkat cells grown in suspension, the protocols were followed, as described (25).

### Confocal immunostaining

Cells were plated on coverslips in 35-mm culture dishes. After treatment, cells were fixed with 2.0% paraformaldehyde for 20 min at room temperature, washed three times for 5 min with 1× phosphate-buffered saline (PBS), permeabilized with 0.2% Triton X-100 in PBS for 10 min and blocked in 3% fetal bovine serum in PBS containing 0.1% Triton X-100 for 1 h. The coverslips were then immunostained using mouse anti-γH2AX (Millipore, Billerica, MA, USA), rabbit anti-53BP1 (Abcam, Cambridge, MA, USA), -phospho-S25-53BP1 and S1778-53BP1 (Cell Signaling Technology, Danvers, MA, USA); mouse anti-phospho-T2609-DNA-PKcs (Abcam), rabbit anti phospho-S2056-DNA-PKcs (Abcam) and -Rad51 (Santa Cruz Biotechnology, Santa Cruz, CA, USA) antibodies, followed by a fluorescently conjugated (Invitrogen) secondary antibody. Mounting and staining of the nuclei were performed using Vectashield-containing 4,6-diamidino-2-phenylindole (Vector Laboratories). Images were collected using an HCX Plan Apo 63×/1.4N.A. oil immersion objective lens on a Leica TCS-SP2 confocal microscope (Leica Microsystems AG). Quantification was based on data observed from 70 cells. Inhibitors of ATM (KU55933) and DNA-PKcs (NU7441) were from Selleck Chemicals (Houston, TX, USA).

### Comet assay

HEK 293T cells were transiently transfected with HA-p18CycE using Lipofectamine 2000 as indicated earlier in the text. At 24 h, posttransfection cells were irradiated with 10 Gy and collected after 16 h. To evaluate the degree of DNA damage, Comet Assay Silver (Trevigen, Gaithersurg, MD, USA) was used, which combines a neutral lysis in low point-melting agarose with silver staining or SYBR Green for visualization of the DNA by single-cell gel electrophoresis. Lysis and electrophoresis were performed according to the manufacturer’s protocol. Image analysis and quantification were performed with NIH ImageJ. Tail Moment (TM) and Tail Length (TL) were used to quantify the DNA damage. TM = % of DNA in the tail × TL; where % of DNA in the tail = tail area (TA) × tail average intensity (TAI) × 100/(TA x TAI) + [head area (HA) × head area intensity (HAI)].

### DNA-agarose pull-down

Nuclear extracts of HEK 293T stably expressing HA-p18CycE were prepared by hypotonic swelling lysis in 20 mM Tris–HCl (pH 8.0) and 1 mM dithiothreitol (DTT). After incubation on ice for 30 min, nuclei were collected by centrifugation at 8000 *g* for 5 min and extracted in 20 mM Tris–HCl (pH 8.0), 500 mM NaCl, 5 mM MgCl_2_, 10% glycerol and 1 mM DTT for 30 min, gently rocking at 4°C. All buffers contained 1× HALT phosphatase inhibitor cocktail (Pierce, Rockford, IL, USA), 1 µg/ml aprotinin, leupeptin, pepstatin and 1 mM phenylmethylsulfonyl fluoride. Extracts were clarified by centrifugation at 16 000*g* for 10 min and diluted (1:3) with 20 mM Tris–HCl (pH 8.0), 5 mM MgCl_2_, 10% glycerol and 1 mM DTT. A slurry (50 µl) of denatured calf thymus DNA-agarose beads (Amersham, Piscataway, NJ, USA) was washed three times, added to the extracts and incubated at 4°C with rotation overnight. Beads were washed three times with 1 ml of buffer, and the proteins were eluted with 500 mM KCl and separated by sodium dodecyl sulphate (SDS) polyacrylamide gel electrophoresis (PAGE), as described (10). The immunoblot was probed with the indicated antibodies.

### DNA end-ligation and plasmid reactivation assay

One micrograms of EcoRI-digested pUC18 DNA (as a surrogate for DSBs) was incubated with nuclear extracts of either p18CycE or CycE-transfected or control (parental or EGFP-expressing), HEK 293 T cells, in reaction buffer X [40 mM Tris–HCl (pH 7.5), 10 mM MgCl_2_, 50 M dNTPs, 2 mM ATP, 1 mM DTT and 100 mg/ml BSA]. The end ligation mixture was incubated at 37°C for 1 h. The product was separated and analyzed by electrophoresis on 0.6% agarose gels. A linearized pUC18 was used as a negative control and, following ligation with T4 DNA ligase, as a positive control. For the plasmid reactivation assay, EcoRI-linearized pUC18 DNA was incubated with nuclear extract of transfected HEK 293T cells in reaction buffer X at 37°C for 1 h. DNA was purified, transformed into DH5 *Escherichia coli*, and cells were plated on Luria Broth (LB) media with ampicillin (Amp) and colonies were scored by a colony counter.

### Chromatin recruitment assay

Irradiated or untreated cells were subjected to chromatin recruitment assay as described (26). Cells were lysed with extraction buffer containing hydroxyethyl piperazineethanesulfonic acid (HEPES) Ethylene diamine tetraacetic acid (EDTA) protease inhibitor, phosphatase inhibitor and triton X-100. Cells were incubated on ice for 20 min, and the pellet was collected after centrifuging at 14 000*g* for 3.5 min. The pellet was incubated at room temperature in a shaker for 30 min after addition of the same extraction buffer, except Triton X-100; RNAse A was included in the buffer. The pellet was collected again after centrifugation at 14 000*g* for 3.5 min. The pellet was suspended in PBS containing 1% SDS. The sample was heated for 10 min and sonicated for 10 s before separation by SDS–PAGE and immunoblotted with the indicated antibodies.

### Statistical analysis

All statistical analyses were done by using two-way ANOVA, and the statistical significance was evaluated for *P* < 0.05.

## RESULTS

### p18CycE generation contributes to decreased cell survival following radiation

**Figure 1.**
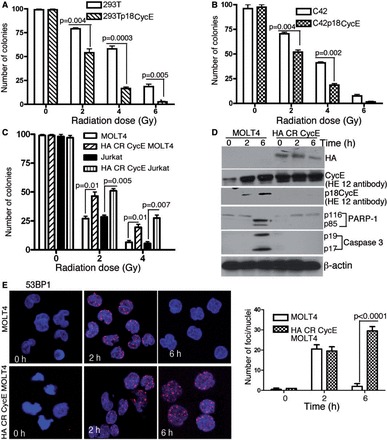
Reduced cell survival and radiosensitivity of p18CycE-expressing cells. (**A** and **B**) Clonogenic cell survival assay of HEK 293T and C4-2 parental and p18CycE-expressing cells following increasing doses of IR. (**C**) Clonogenic cell survival of Jurkat and MOLT4 and derivative CR CycE-expressing cells following irradiation. (**D**) Western blot showing the expression of HA CR CycE as well as the endogenous levels of p18CycE, full-length CycE and cleaved PARP-1 and Caspase 3. β-actin was used as loading control. (**E**) Confocal immunostaining (right) for 53BP1 in MOLT4 and CR CycE-expressing cells following radiation at the indicated time points. Graphical representation (left panel) of 53BP1 IRIFs following radiation. Error bars represent SD (*n* = 3).

Two different epithelial tumor cell types, kidney (HEK 293T) and prostate (C4-2), were used for stable ectopic expression of p18CycE, as p18CycE is not produced endogenously in these cells. There was a significant IR dose-dependent (0–6 Gy) reduction in clonogenic survival in p18CycE-expressing HEK 293T compared with the parental cells (Figure 1A). Similarly, there was a significantly reduced cell survival of p18CycE-expressing C4-2 prostate cancer cells compared with the parental cells following IR (Figure 1B). Levels of p18CycE, expressed stably in HEK 293T and C4-2 derivative cells (Supplementary Figure S1D), were comparable with those of endogenous CycE in HEK 293T parental and p18CycE-derivative cells (Supplementary Figure S1D, left panel).

We next examined the physiological relevance of p18CycE generation for DNA repair in leukemic cells. First, clonogenic cell survival was performed in response to IR in MOLT4 cells, in which p18CycE is generated endogenously following IR (21,27), and derivative cells, stably expressing a cleavage-resistant (CR) form of CycE. Parental MOLT4 cells were more sensitive to radiation compared with CR CycE-expressing cells (Figure 1C), as significantly reduced numbers of colonies were observed with an increased dose of radiation. Similar results were obtained in Jurkat cells (Figure 1C). Ectopic expression of CR CycE diminished IR-induced cell death in MOLT4 as indicated by western blotting, as described (21,27) for cleaved PARP-1 and caspase 3 (Figure 1D). Importantly, MOLT4 cells showed diminished 53BP1 IRIFs at 6 h following IR, whereas CR CycE-expressing cells maintained 53BP1 IRIFs (Figure 1E), indicating that DNA repair was impaired following p18CycE generation in MOLT4 cells. Taken together, these findings indicate that the presence of p18CycE renders the cells radiosensitive, as reflected by their decreased 53BP1 IRIFs and cell survival.

### p18CycE inhibits *in vitro* DNA ligation and plasmid reactivation

**Figure 2.**
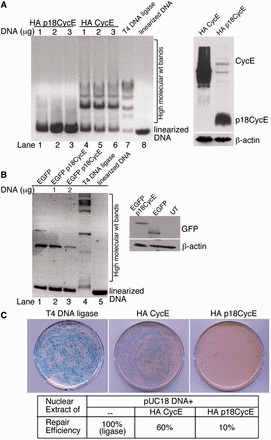
p18CycE inhibits *in vitro* DNA ligation and plasmid reactivation. (**A**) Nuclear cellular extracts from HEK 293T transfected with increasing amounts of HA p18CycE (lanes 1–3) and HA CycE (lanes 4–6) were incubated with a pUC18 plasmid DNA linearized with the EcoRI restriction enzyme. The reaction was incubated at 37°C to allow the DNA to be re-ligated. T4 DNA ligase reaction and linearized pUC18 were used as controls (lane 7–8). The DNA ligation reaction was separated by gel electrophoresis. Expression of the transfected constructs was determined by immunoblotting with anti-HA antibody (right panel). (**B**) Nuclear cellular extracts from HEK 293T cells transfected with EGFP or EGFP p18CycE, or untransfected (UT) were used as described earlier in the text (A) for *in vitro* DNA ligation. Lanes 2 and 3 (left panel) represent identical reactions. The right panel shows the expression of EGFP and EGFP p18CycE immunoblotted with anti-EGFP antibody. β-actin was used as a loading control. (**C**) The DNA ligation reaction mix was transformed into *E. coli* that were then plated on agar plates containing Amp. The number of colonies was expressed relative to the percentage of those obtained with the T4 DNA ligase-treated DNA that were considered to be 100%.

As p18CycE can bind to Ku70 (22), we next examined whether it is capable of interfering with DNA repair using a biochemical *in vitro* DNA end-ligation assay. In the presence of full-length CycE (lanes 1–3) or T4 DNA ligase, used as a positive control (lane 7), the pUC18 plasmid DNA was efficiently re-ligated, as indicated by its higher molecular weight circular, monomeric or concatemeric forms (Figure 2A, left panel). In contrast, in the presence of HA p18CycE (lanes 4–6), the plasmid DNA remained in its linear form. Expression of comparable p18CycE and CycE levels was confirmed by immunoblotting with anti-HA antibodies (Figure 2A, right panel). These findings were confirmed by using another construct in an independent experiment (Figure 2B). Expression of EGFP p18CycE also blocked end-joining (lane 2–3), as indicated by the absence of the higher molecular weight forms present in EGFP-expressing cells (lane 1). Comparable expression of the transfected constructs was validated by western blot (Figure 2B, right panel). These results suggest that p18CycE inhibited the plasmid re-ligation as observed by the absence of higher molecular weight DNA forms. Moreover, the ability of the re-ligated plasmid to form Amp-resistant colonies (indicating productive re-ligation) following transformation in *E. coli* was dramatically reduced in the presence of p18CycE to ∼10%, as compared with ∼60% for CycE or T4 DNA ligase, considered to be 100% (Figure 2C). These data suggest that p18CycE inhibits end-joining in these two NHEJ DNA repair biochemical assays.

### p18CycE expression increases DNA damage following irradiation

**Figure 3.**
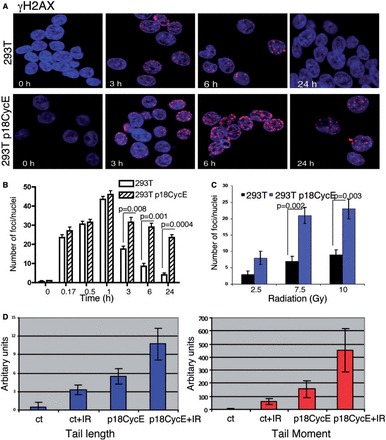
p18CycE expression augments DNA damage induced by IR. (**A**) Confocal immunostaining for γH2AX in HEK 293T and p18CycE-expressing derivative cells at the indicated times following radiation. (**B**) Graphical representation for γH2AX foci after radiation at different time points (left panel) and at different doses (right panel; 24 h) in HEK 293T parental and p18CycE-expressing cells. (**C**) Comet assay, indicating TL (left panel) and TM (right panel) in HEK 293T cells and those transiently expressing p18CycE following radiation. Error bars represent SD (*n* = 3). Ct, control.

As p18CycE can inhibit end-joining, we next examined whether it affects cellular DNA damage responses. Radiation produces DNA breaks that can be assessed by scoring the number of γH2AX foci, a surrogate marker for DNA damage and its repair. After radiation treatment, γH2AX IRIFs accumulated in HEK 293T and derivative p18CycE-expressing cells for at least 1 h. p18CycE-expressing, but not parental HEK 293T cells, showed persistent γH2AX IRIFs at 24 h (Figure 3A and B). As expected, increasing the radiation dose increased the number of γH2AX foci in both cell lines; however, p18CycE-expressing cells displayed significantly elevated numbers of foci (Figure 3C). Up to 1 h following IR, the γH2AX IRIFs indicated similar levels of DNA damage in both parental and p18CycE-expressing cells. However, starting at 3 h following IR, the γH2AX IRIFs were significantly increased in p18CycE-expressing cells (Figure 3B). In contrast, HEK 293T cells expressing HA CycE did not differ in the number or kinetics of γH2AX IRIFs compared with parental cells, with foci being prominent following 1 h of radiation and resolved by 6 h (Supplementary Figure S1A).

Another sensitive assay to detect DNA damage is the comet assay, which measures broken DNA and fragments, scored as TM and TL (28). TL and TM increased slightly following transfection with p18CycE. Importantly, in these cells, transiently expressing p18CycE, both TL and TM were further increased following IR (Figure 3D), indicating prolonged DNA damage, which in turn suggests inefficient repair of DNA breaks. Similar results were obtained using a DNA damage-inducing chemotherapeutic agent CPT-11 (data not shown).

### Retention of NHEJ components on chromatin is reduced in p18CycE-expressing cells

**Figure 4.**
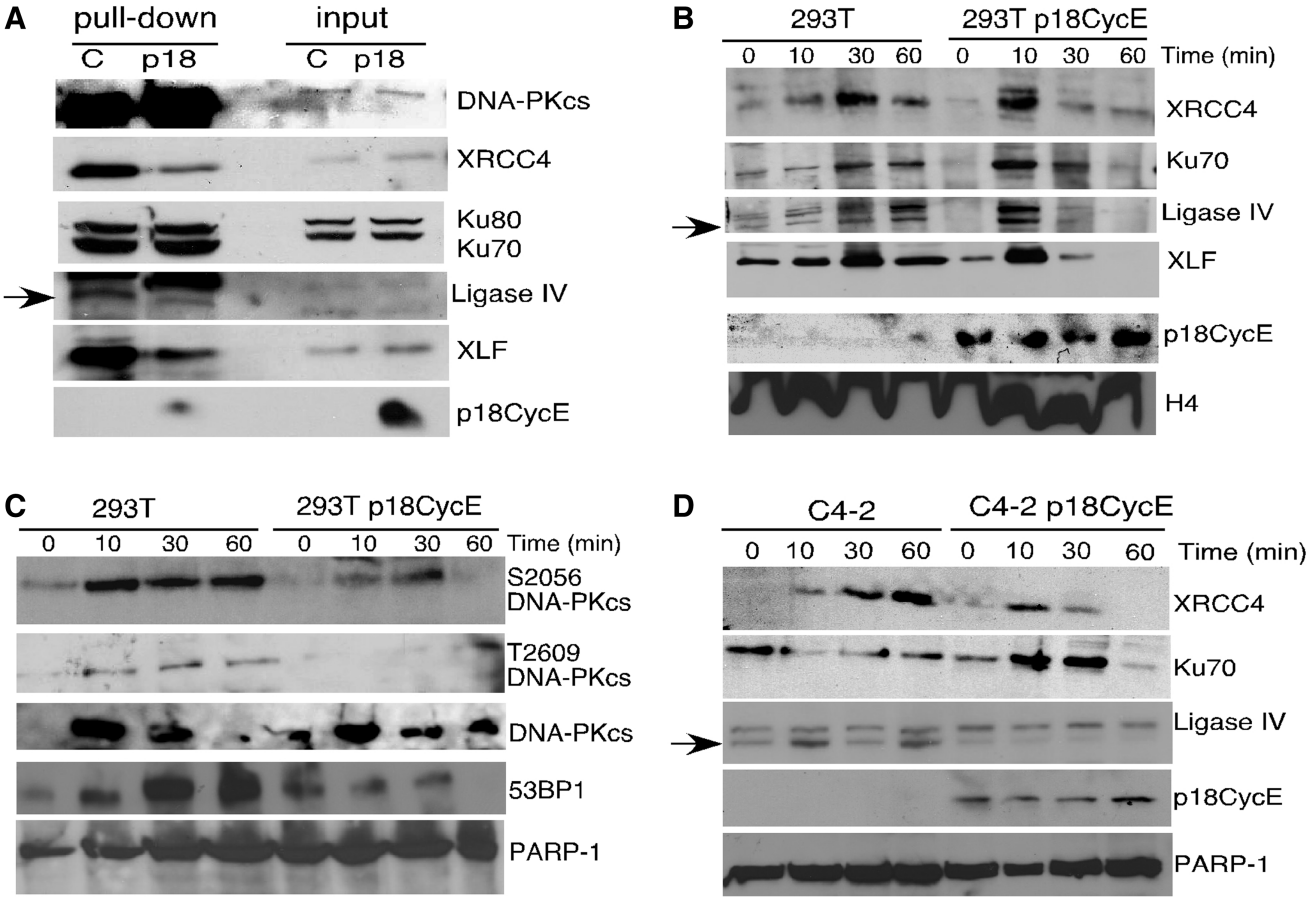
p18CycE interferes with chromatin retention of NHEJ components. (**A**) Nuclear extracts of HEK 293T cells stably expressing p18CycE or EGFP were used for determining the levels of NHEJ proteins, XRCC4, Lig IV, XLF, Ku70-80 and DNA-PKcs recruited to DNA-conjugated beads. Input was used to indicate equal protein lysates. Time-course for recruitment of NHEJ proteins to the chromatin fraction of parental and p18CycE-expressing derivative (**B**) HEK 293T kidney and (**D**) C4-2 prostate cancer cells. (**C**) DNA-PKcs autophosphorylation and levels of DNA-PKcs and 53BP1 in the chromatin-bound fraction of HEK 293T parental and p18CycE-expressing derivative cells. Histone H4 and PARP-1 were used as loading controls.

Following generation of DNA DSBs, Ku70/Ku80 heterodimers bind the free DNA ends to keep them in close proximity and provide a platform for recruitment of other critical DNA repair factors, such as DNA-PKcs, Artemis, XRCC4, XLF and Ligase IV (8). To determine at which step in the NHEJ pathway p18CycE acts, a chromatin recruitment assay was used based on nuclear extracts from HEK 293T cells expressing p18CycE or EGFP, as a control. DNA DSBs were mimicked by denatured calf thymus DNA fragments cross-linked to agarose beads. The DNA repair complexes were allowed to form at the ends of the DNA fragments and then were eluted with high saline buffer and separated by SDS–PAGE. Interestingly, the binding of the Ku70/Ku80 heterodimer to the DNA fragments was not affected. In contrast, the recruitment of the XLF/XRCC4/Ligase IV heterocomplex was dramatically reduced in the presence of p18CycE (Figure 4A), suggesting its interference with the last step in NHEJ, the ligation of the DNA ends. Moreover, p18CycE was also bound to the DNA fragments. Surprisingly, the recruitment/retention of DNA-PKcs to DSBs appeared to be enhanced.

We next examined chromatin recruitment of NHEJ components in intact parental and p18CycE-expressing HEK 293T and C4-2 cells following IR. Ku70, XRCC4, Ligase IV, XLF and DNA-PKcs were recruited to chromatin as early as 10 min postirradiation (Figure 4B). However, by 1 h, Ku70, XRCC4, Ligase IV and XLF were largely dissociated from the chromatin in p18CycE-expressing cells. In contrast, in HEK 293T and C4-2 parental cells, these critical NHEJ proteins remained on the chromatin (Figure 4B and D). Moreover, in p18CycE-expressing cells, the chromatin-bound fraction showed diminished phosphorylation of DNA-PKcs on T2609 and S2056, after IR, compared with parental cells. This is consistent with the higher retention and therefore more abundant DNA-PKcs on the chromatin in p18CycE-expressing cells (Figure 4C). Surprisingly, p18CycE was constitutively bound to chromatin (Figure 4B and D). 53BP1, another critical protein for coupling the DNA damage response to DNA repair, also detached from the chromatin in p18CycE-expressing, but not parental, HEK 293T cells by 1 h further suggesting that the DNA repair was suppressed (Figure 4C).

### Autophosphorylation of DNA-PKcs is inhibited in p18CycE-expressing cells

**Figure 5.**
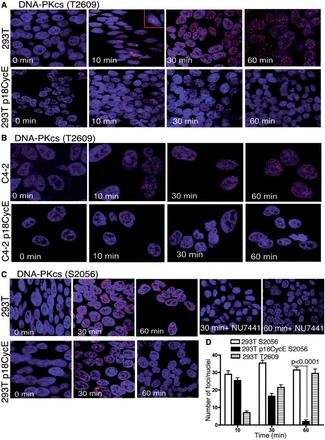
p18CycE expression prevents DNA-PKcs autophoshorylation. Confocal immunostaining for T2609-DNA-PKcs phosphorylation following irradiation in parental and p18CycE-expressing (**A**) HEK 293T and (**B**) C4-2 cells. (**C**) Confocal immunostaining of S2056 phosphorylation of DNA-PKcs following radiation in HEK 293T and p18CycE-expressing cells. (**D**) Graphical representation of the number of foci per cell at the indicated time for the two DNA-PKcs phosphorylation sites in HEK 293T parental and p18CycE-expressing cells. Error bars represent SD (*n* = 3).

DNA-PKcs, the critical kinase for NHEJ, is recruited by the Ku70/80 heterodimer to DSB sites. Its autophosphorylation on T2609 (in the ABCDE cluster) and S2056 (in the PQR cluster) leads to a conformational change, which in turn regulates its kinase activity and ultimately leads to its dissociation from the DNA ends. T2609 was phosphorylated as early as 10 min and retained up to 60 min following IR-induced DNA damage in HEK 293T and C4-2 parental cells. In contrast, T2609 phosphorylation was completely suppressed in p18CycE-expressing cells (Figure 5A and B), consistent with the impaired dissociation of DNA-PKcs from the chromatin (Figure 4D).

Autophosphorylation of S2056-DNA-PKcs was IR dose-dependent. It reached a peak at 30 min and was persistent for at least 1 h after IR in HEK 293 T parental cells. Surprisingly, in p18CycE-expressing cells, this phosphorylation was diminished by 30 min following IR (Figure 5C), again consistent with impaired NHEJ repair. Inhibition of DNA-PKcs by the pharmacological inhibitor, NU7441, abrogated the phosphorylation of S2056-DNA-PKcs (Figure 5C). The graphical representation of IRIFs dynamics following IR clearly shows a significant difference between the two cell lines at both of these different autophosphorylation sites (Figure 5D).

### p18CycE expression reduces the number of 53BP1 IRIFs

**Figure 6.**
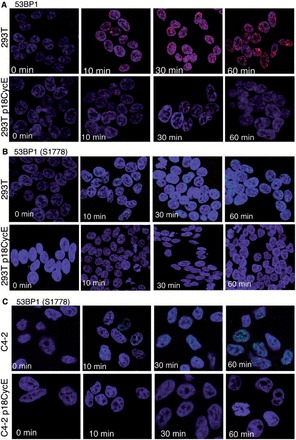
p18CycE expression reduces the number of 53BP1 foci. (**A**) Confocal immunostaining for total 53BP1 in HEK 293T parental and p18CycE-expressing cells following radiation. Confocal immunostaining for S1778 phosphorylation of 53BP1, a target of DNA-PKcs in (**B**) HEK 293T parental and p18CycE-expressing cells and in (**C**) C4-2 and p18CycE-expressing cells following radiation.

After its chromatin recruitment, DNA-PKcs phosphorylates a number of proteins critical for NHEJ (27), including 53BP1. Examination of IRIFs formed by 53BP1 and its phosphorylated form revealed that the number of 53BP1 foci increased in a time-dependent manner up to 60 min after IR in parental HEK 293T cells. In p18CycE-expressing cells, the total number of IRIFs was very low compared with the parental cells (Figure 6A and Supplementary Figure S2C). Moreover, phosphorylation of the DNA-PKcs phosphorylation site on 53BP1 (S1778) (13) was abolished in both p18CycE-expressing HEK 293T and C4-2 cells, in sharp contrast to the parental cells (Figure 6B and C). Decreased 53BP1 IRIFs were consistent, as found in both MOLT4 cells endogenously producing p18CycE following IR (Figure 1D) and in HEK 293T cells ectopically expressing it (Figure 6A). Moreover, confocal immunostaining for 53BP1 in HEK 293T cells, expressing full-length HA CycE, showed 53BP1 IRIFs kinetics similar to parental cells (Figure 6A, Supplementary Figure S1B), confirming that p18CycE expression was responsible for suppressing 53BP1 foci. The expression of HA CycE was validated by western blot (Supplementary Figure S1C).

### p18CycE-expressing cells have diminished S25-53BP1 and pMDC1 phosphorylation, but intact ATM signaling

**Figure 7.**
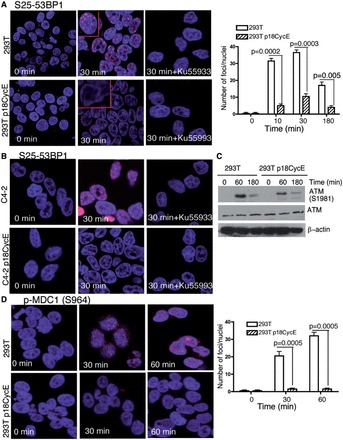
p18CycE blocks MDC1 phosphorylation and suppresses S25-53BP1, but not ATM phosphorylation. (**A**) Confocal immunostaining (left panel) graphical representation (right panel) for phosphorylation of S25-53BP1, which is known to be ATM dependent, at different time points following irradiation of HEK 293T parental and p18CycE-expressing cells in the absence or presence of the ATM inhibitor, KU55933. Error bars represent SD (*n* = 3). (**B**) Confocal immunostaining for S25-53BP1 in C4-2 parental and p18CycE-expressing cells following radiation at 30 min ± the ATM inhibitor KU55933. (**C**) ATM (total and phospho-S1981) expression following irradiation in HEK 293T parental and p18CycE-expressing cells; β-actin was used as loading control. (**D**) Confocal immunostaining (left panel) and graphical representation (right panel) for phosphorylation of MDC1 following radiation at the indicated time points in HEK 293T parental and p18CycE-expressing cells.

p18CycE-expressing cells showed a dramatic decrease in detectable phosphorylation of 53BP1 at S25, which is known to be dependent predominantly on ATM and less on DNA-PKcs (13). Following IR, the number of IRIFs was reduced significantly in p18CycE-expressing HEK 293T cells compared with parental cells (Figure 7A). Pharmacological inhibition of ATM with KU55933 revealed, indeed, that S25-53BP1 phosphorylation was ATM-dependent in these cells as IRIFs of S25-53BP1 were abolished in both parental, as well as p18CycE-expressing cells (Figure 7A). These observations were further confirmed in C4-2 cells, which showed robust S25-53BP1 IRIFs at 30 min following IR, in contrast to p18CycE-expressing cells, where these IRIFs were hardly detectable (Figure 7B). Thus, pharmacological inhibition of ATM completely abrogated IRIF formation in both parental and p18CycE-expressing cells, indicating the ATM dependence for this phosphorylation event.

ATM, an essential sensor of IR-triggered DNA damage response pathway, phosphorylates 53BP1 at the S25/29 site. As S25/29 53BP1 IRIFs diminished significantly in p18CycE-expressing cells, we next investigated ATM phosphorylation. At 60 and 180 min following IR, both parental and p18Cyc-E-expressing cells showed the activated phosphorylated S1981-ATM form, with comparable ATM protein levels (Figure 7C). A previous study (29) suggested that 53BP1 foci alone do not account for the S1981 phosphorylation of ATM. This study further showed that MDC1, a critical mediator of DNA damage response, is an upstream regulator of S25/29 phosphorylation of 53BP1. Analyses of S964-MDC1 IRIFs following radiation in p18CycE-expressing cells revealed, indeed, diminished foci at 30 and 60 min following IR (Figure 7D). These results indicate that although ATM activation is not affected by p18CycE expression, phosphorylation of its downstream mediators, MDC1 and 53BP1, is repressed.

## DISCUSSION

Identification of interactions between Ku70, a key NHEJ core component, and the radiation-induced apoptosis-generated proteolytic fragment of Cyclin E (p18CycE) (22,24,30) provided the impetus for exploring the role of p18CycE in NHEJ DNA repair. In leukemic cells, where p18CycE is generated endogenously following IR, it has a profound effect on cell survival. Clonogenic assays performed in leukemic MOLT4 and Jurkat cells showed radiosensitivity at different doses of IR. This sensitivity to radiation could be overcome by overexpressing a CR form of CycE (CR CycE), leading to significant increase in colony survival following IR compared with parental MOLT4 or Jurkat cells. In epithelial tumor cells, in which p18CycE is not generated endogenously, its stable expression induces autophagy and senescence, rather than apoptosis in response to IR (27). The expression of p18CycE acutely or chronically (21,22,24) confers radiosensitivity to tumor cells, thus providing the rationale for investigating the impact of p18CycE expression on NHEJ DNA repair functions.

Several reports suggest that mutations in DNA-PKcs, at either the T2609 or S2056 autophosphorylation sites, can produce mild radiosensitivity, whereas the combined mutations act synergistically to render these cells more radiosensitive (20,31). Indeed, phosphorylation at both S2056 and T2609 sites has been reported to be important for DNA-PKcs-mediated radioresistance (17). In cells containing the T2609 mutant DNA-PKcs, the resolution of γH2AX was delayed considerably following IR compared with the DNA-PKcs null cells (19), indicating sustained DNA damage (32). Similarly, chronic expression of p18CycE sensitized epithelial cells to radiation. Both neutral comet assays and γH2AX foci formation, two DSB indicators, showed marked differences in the repair kinetics of p18CycE-expressing cells. Moreover, T2609 and S2056-DNA-PKcs IRIFs were greatly diminished in the presence of p18CycE, indicating an impact on normal NHEJ function. IRIFs for the DNA damage marker 53BP1, which is known to promote NHEJ, were significantly abolished in MOLT4 cells at 6 h following IR, when p18CycE was endogenously generated (Figure 1D). Similar observations were made for HEK 293T cells ectopically expressing p18CycE, as 53BP1 IRIFs were significantly reduced compared with parental cells.

At the sites of DSBs, the Ku70/80 heterodimers bind to the chromatin to initiate NHEJ by recruiting DNA-PKcs. The Ku70/80 heterodimeric complex translocates inwards to create a platform for XRCC4 and XLF binding to DNA, allowing DNA ligation by activating ligase IV (33). Chromatin recruitment at DSBs of Ku70-Ku80, DNA-PKcs, XRCC4, ligase IV and XLF was largely unaffected in p18CycE-expressing cells. However, their retention was diminished in the presence of p18CycE. Moreover, the activity of DNA-PKcs was suppressed in the presence of p18CycE, as indicated by the inability to form IRIFs by DNA-PKcs (T2609 and S2056), 53BP1 and S1778-53BP1 (known to be a substrate of DNA-PKcs) (13), to an extent similar to its pharmacological inhibition by NU7441. Strikingly, DNA-PKcs dissociation from DSBs was impaired, consistent with defective autophosphorylation at T2609 and S2056 in the presence of p18CycE. These data are consistent with the interpretation that the presence of p18CycE on the chromatin interferes with NHEJ, making the p18CycE-expressing cells more radiosensitive. A recent report (34) suggests that Ligase IV contributes to DNA-PKcs autophosphorylation, and end-joining occurs by early formation of a supramolecular entity containing DNA-PK, XLF and Ligase IV complexes on DNA ends. In p18CycE-expressing cells, both XLF and Ligase IV retention was defective along with diminished DNA-PKcs autophosphorylation, suggesting that p18CycE is perturbing the formation of the end-joining complex. As a consequence of decreased retention of Ligase IV and XLF, DNA repair was also defective.

Earlier studies have indicated that the DNA-PKcs autophosphorylation status determines whether a cell chooses to repair DSBs by HR or NHEJ (19). When the ABCDE cluster phosphorylation is defective, HR is blocked. However, subsequent deficient PQR cluster phosphorylation enhances HR (19), thus suggesting that cells lacking functional DNA-PKcs suppressed phosphorylation at both the T2609 and S2056 sites have augmented HR. Rad51 is a key participant in HR after radiation that has been reported to compensate for diminished NHEJ (19). Indeed, evidence for HR could be detected via elevated RAD51 foci much earlier after radiation in p18CycE-expressing cells compared with parental cells. There was increased localization of HR proteins to damaged chromatin by 1 h following IR in p18CycE-expressing, compared with parental, cells while NHEJ was impaired, as indicated by a block in DNA-PKcs autophosphorylation on T2609 and S2056 (Supplementary Figure S2A and B).

In contrast to DNA-PKcs, another DNA damage sensor, ATM, was not directly affected by the presence of p18CycE, as there was no difference in ATM phosphorylation in parental and p18CycE-expressing cells. However, there was a significant defect in S25/29 phosphorylation of 53BP1, which is known to be ATM mediated. Moreover, phosphorylation of the mediator protein, MDC1, was also suppressed in p18CycE-expressing cells. A previous report (29) has shown that MDC1 is responsible for phosphorylation of 53BP1 at S25/29 site even when ATM is active, and that defective MDC1 or diminished 53BP1 is not sufficient to inhibit ATM activation. Similarly, we could not find any defect in ATM activation in p18CycE-expressing cells. However, at least for two of its targets, S964-MDC1 and S25 53BP1, phosphorylation was suppressed.

In summary, these findings reveal a unique biological circumstance, in which the presence of a proteolytic fragment of CycE perturbs the association of NHEJ factors at the site of DSBs on the chromatin following irradiation, resulting in decreased cell survival. p18CycE, originally identified as a binding partner of Ku70, interferes with DNA-PKcs function by inhibiting its autophosphorylation or phosphorylation of its targets, such as S1778-53BP1. Generation of p18CycE following radiation or any other chemotherapeutic agent that induces apoptosis, in all leukemic cells we have examined, could have a significant effect on DNA damage repair, as well as cell survival. These findings uncover a novel mechanism for inactivation of NHEJ that can impact cell survival.

## SUPPLEMENTARY DATA

Supplementary Data are available at NAR Online.

## FUNDING

National Institutes of Health (NIH), [CA127264 to A.A.] and by the US Department of Energy Office of Science under contract number [DE-AC02-05CH11231 to S.M.Y.]. Funding for open access: NIH [CA127264].

*Conflict of interest statement*. None declared.

## Supplementary Material

Supplementary Data
