# Supplementary material for: Defective chromatin recruitment and retention of NHEJ core components in human tumor cells expressing a Cyclin E fragment
Source: Nucleic Acids Res. 2013 Sep 9;41(22):10157–69. doi: 10.1093/nar/gkt812 (PMC3905870; doi:10.1093/nar/gkt812)
Supplement: Supplementary Data [file supp_41_22_10157__index.html]

Defective chromatin recruitment and retention of NHEJ core components in human tumor cells expressing a Cyclin E fragment — Defective chromatin recruitment and retention of NHEJ core components in human tumor cells expressing a Cyclin E fragment — Supplementary Data 

# Defective chromatin recruitment and retention of NHEJ core components in human tumor cells expressing a Cyclin E fragment

## Supplementary Data

files

**Files in this Data Supplement:**

- Supplementary Data - doc file
- Supplementary Data - jpg file
- Supplementary Data - jpg file
