# Supplementary material for: Defective chromatin recruitment and retention of NHEJ core components in human tumor cells expressing a Cyclin E fragment
Source: Nucleic Acids Res. 2013 Sep 9;41(22):10157–69. doi: 10.1093/nar/gkt812 (PMC3905870; doi:10.1093/nar/gkt812)
Supplement: Supplementary Data [file supp_gkt812_nar-00149-d-2013-File009.doc]

**Supplementary Figure 1. Full length CycE overexpression does not affect DNA damage repair.** (**A**) & (**B**) Confocal immunostaining for γH2AX and 53BP1 foci, respectively following radiation at indicated time points, in HEK 293T cells expressing HA CycE. (**C**) Immunoblot probed with anti-HA to validate the ectopic expression of full length HA CycE in HEK 293T cells, in contrast to parental cells. β-actin was used as loading control. Western blot showing the levels of p18CycE expressed stably in HEK 293T and C4-2 cells, compared to parental cells and (**D**) produced endogenously in Jurkat cells following IR (**E**). β-actin was used as loading control.

**Supplementary Figure 2. p18CycE-expressing cells show elevated Rad51 and 53BP1 foci.** (**A**) Confocal immunostaining and (**B**) graphical representation for Rad51 following radiation at indicated time points in HEK 293T parental and p18CycE-expressing cells. (**C**) Graphical representation for 53BP1 IRIFs in HEK 293T parental and p18CycE-expressing cells (immunostaining Figure 6A).
