# Supplementary figures and images for: Defective chromatin recruitment and retention of NHEJ core components in human tumor cells expressing a Cyclin E fragment
Source: Nucleic Acids Res. 2013 Sep 9;41(22):10157–69. doi: 10.1093/nar/gkt812 (PMC3905870; doi:10.1093/nar/gkt812)

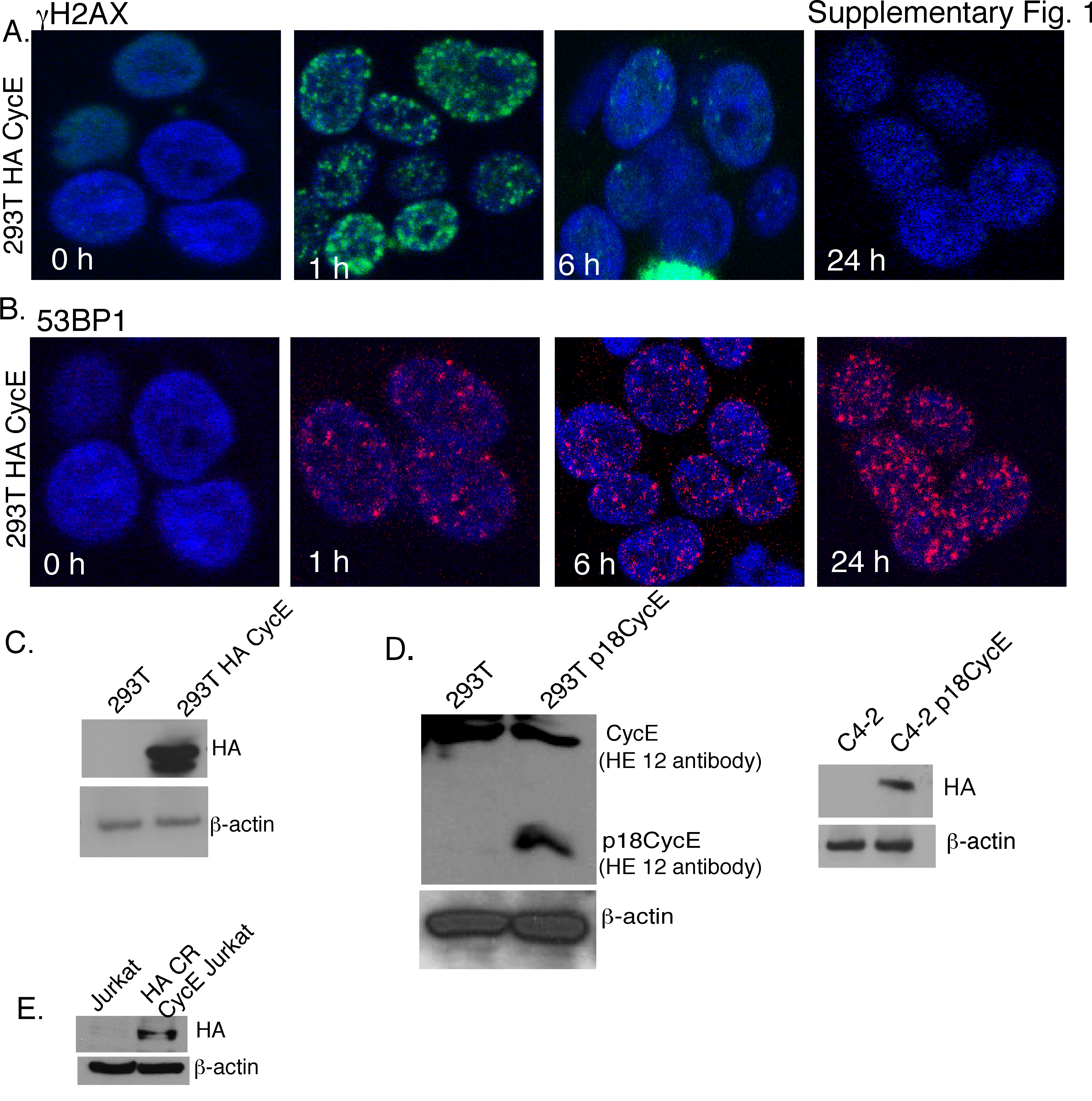

Supplement: Supplementary Data [file supp_gkt812_nar-00149-d-2013-File010.jpg]

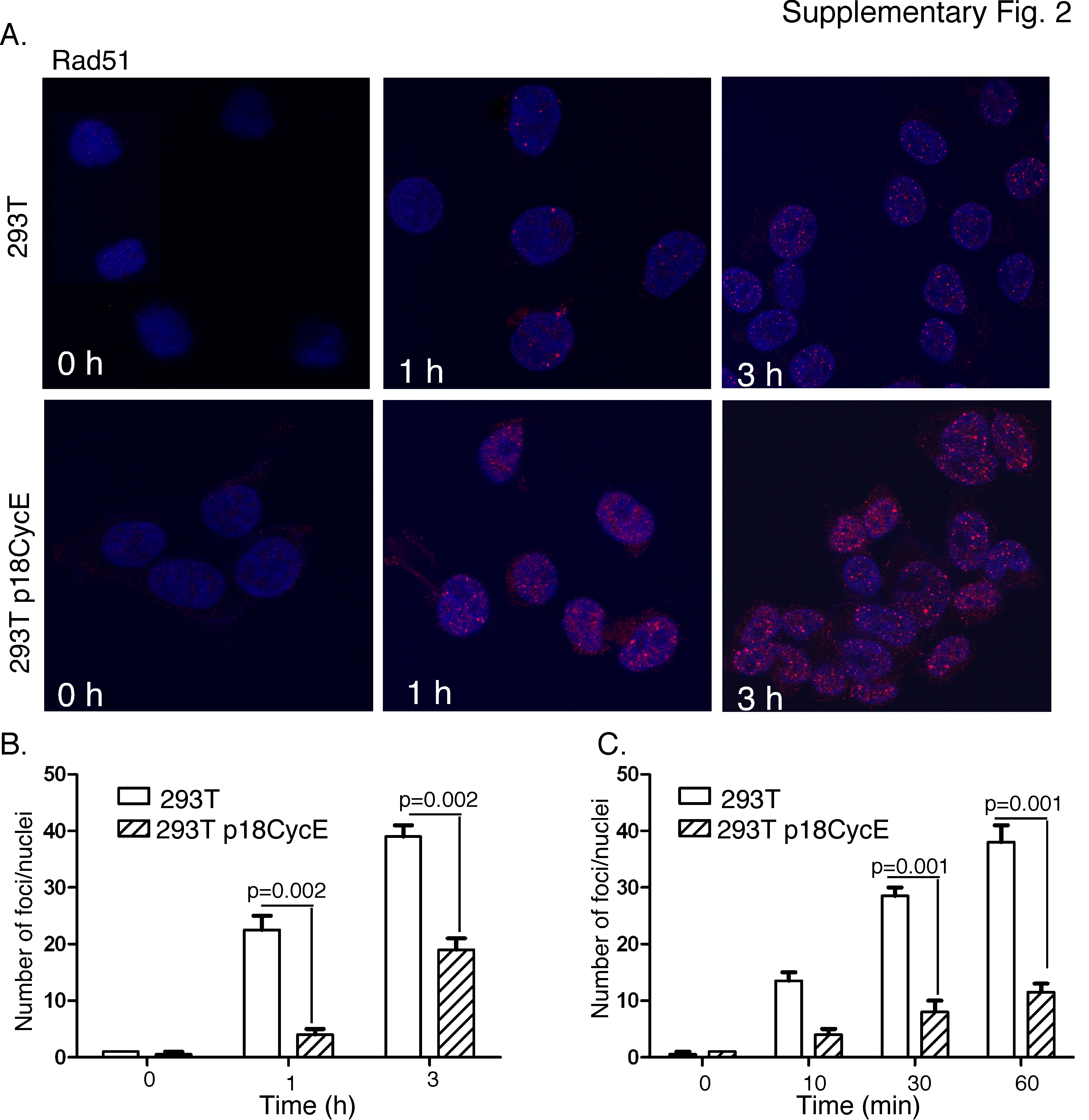

Supplement: Supplementary Data [file supp_gkt812_nar-00149-d-2013-File011.jpg]
